# Knowledge Transfer and Exchange Processes for Environmental Health Issues in Canadian Aboriginal Communities

**DOI:** 10.3390/ijerph7020651

**Published:** 2010-02-23

**Authors:** Susan M. Jack, Sandy Brooks, Chris M. Furgal, Maureen Dobbins

**Affiliations:** 1 School of Nursing, McMaster University, 1200 Main Street West, Hamilton, ON L8N 3Z5, Canada; E-Mails: sbrooks@mcmaster.ca (S.B); dobbinsm@mcmaster.ca (M.D.); 2 Indigenous Environmental Studies, Trent University, 1600 West Bank Drive, Peterborough, ON K9J 7B8, Canada; E-Mail: chrisfurgal@trentu.ca (C.M.F.)

**Keywords:** environmental health, research utilization, knowledge transfer, Aboriginal, qualitative

## Abstract

Within Canadian Aboriginal communities, the process for utilizing environmental health research evidence in the development of policies and programs is not well understood. This fundamental qualitative descriptive study explored the perceptions of 28 environmental health researchers, senior external decision-makers and decision-makers working within Aboriginal communities about factors influencing knowledge transfer and exchange, beliefs about research evidence and Traditional Knowledge and the preferred communication channels for disseminating and receiving evidence. The results indicate that collaborative relationships between researchers and decision-makers, initiated early and maintained throughout a research project, promote both the efficient conduct of a study and increase the likelihood of knowledge transfer and exchange. Participants identified that empirical research findings and Traditional Knowledge are different and distinct types of evidence that should be equally valued and used where possible to provide a holistic understanding of environmental issues and support decisions in Aboriginal communities. To facilitate the dissemination of research findings within Aboriginal communities, participants described the elements required for successfully crafting key messages, locating and using credible messengers to deliver the messages, strategies for using cultural brokers and identifying the communication channels commonly used to disseminate and receive this type of information.

## 1. Introduction

Environmental health policy is developed to protect health and human well-being and to reduce preventable injuries and diseases caused by physical, chemical, social, psychosocial or biological hazards in natural and man-made environments [1]. The development of environmental health policies is a highly political process and policy outcomes have diverse and varied impacts on individuals, groups, economic interests and geographic regions. Globally there is increasing momentum to utilize research evidence derived from Western scientific methods and to adopt evidence-informed policy development processes in lieu of opinion-based policy [2]. It has been documented however that there are significant time lags between the points of knowledge creation and its utilization in decision-making [3].

In the adoption of evidence-informed decision-making there is an interesting paradox in that different stakeholder groups have unique definitions of what constitutes evidence. Researchers tend to define evidence as knowledge systematically developed using the scientific process whereas individuals responsible for practice, managerial or policy decisions, more broadly define evidence to include scientific research and locally relevant information [4]. Many Aboriginal environmental health issues are interesting case studies in this regard as decision-makers in public and private sectors have worked to develop strategies for integrating both research evidence and Traditional Knowledge. This has occurred particularly in regards to conservation, land and resource management, and environmental assessment projects [5–7]. Ellis [5] defines Traditional Knowledge as “a cumulative, collective body of knowledge, experience, and values held by societies with a history of subsistence” (p. 66). In the literature, Traditional Knowledge may also be referred to as Traditional Aboriginal knowledge, Traditional Indigenous knowledge or more specifically First Nations Traditional Ecological knowledge (TEK) [8] and Inuit Qaujimajatuqangit [9] referring to Inuit knowledge about the land and environment.

With increasing health system demands for cost containment, accountability and quality improvement, there has been a paradigm shift to identifying strategies to incorporate more research evidence into the policy development process [4]. A similar evolution has taken place in the context of environmental health. The environmental health research process requires a significant investment of human and capital resources. With these investments, researchers and funding agencies have an expectation that research findings will be accessed and appraised by practice and policy decision-makers and used to inform decisions to subsequently improve health outcomes [10]. This activity has increasingly become known as knowledge transfer and exchange (KTE). In general it has been defined as a collaborative and interactive process that incorporates the interchange of different types of knowledge between researchers and decision-makers [11].

Moving research evidence into use in decision-making processes is a complex process. Barriers to implementing evidence-informed decision-making exist at both individual and organizational levels and may include: a lack of time to access review or appraise evidence; limited access to research evidence; poor critical appraisal skills; unsupportive organizational cultures; limited ability to make independent decisions and; resistance to change [12–14]. Similar barriers to KTE were found in a mixed methods study conducted to explore the perceptions of health care providers and policy makers working in Aboriginal health care settings; unique barriers specific to this context included: (1) a perceived lack of trust in researchers; (2) overexposure to research in Aboriginal communities resulting in a perceived desensitization to research findings and; (3) poor formal infrastructures to link decision-makers with researchers who have the skills to assist in answering their research questions [15].

To promote the movement of research evidence into decision-making, it is argued that researchers increasingly have a responsibility to identify more effective strategies for communicating study results to a variety of target audiences, to learn more about the process by which program and policy level decisions are made by administrators and bureaucrats, and to focus on building collaborative relationships with key stakeholders [10,16,17]. As the science of KTE evolves, two groups of actors are normally referred to: (1) researchers or the producers of knowledge and; (2) decision-makers or the knowledge consumers who will adapt the information to inform the development of interventions, programs or policies. Wingens [18] refers to this in the ‘two-communities’ theory where researchers and decision-makers work and function in different cultures with distinct and sometimes conflicting values, beliefs, norms, ways of thinking, language and knowledge. The cultural differences between the two unique environments are often used as a rationale to explain the non-utilization of research evidence in decision-making processes. Following the passing of the Federal Indian Act in Canada, this two-community model was the norm with health researchers and policy makers, generally funded or employed by the Federal government, conducting research within First Nations reserves or other Aboriginal communities [19]. However, in the current context of increased self-determination and the reality of Aboriginal self-governance, increasingly more health policy makers are sought and employed internally within Aboriginal Band Councils, Health Boards, or Regional Inuit Organizations. This has essentially created three communities of actors in the KTE process and adds to the increasing complexity of the KTE process.

One of the goals of the National Collaborating Centre for Aboriginal Health (NCCAH), which is funded through the Public Health Agency of Canada, is to support the development of public health practices and policies through knowledge transfer and knowledge exchange (www.nccah-ccnsa.ca). To achieve this goal, it is critical to identify and understand the sources and types of knowledge, and the various communication channels that are valued and utilized by both the researcher and decision-making communities within Canadian Aboriginal health settings. The interplay between research evidence, often developed and guided based on the perspectives of non-Aboriginal researchers, funding agencies and generated through the scientific method, and Aboriginal Traditional Knowledge and their influences on policy development are currently not well understood. The purpose of this naturalistic qualitative study was to: (1) describe the factors influencing the KTE process between environmental health researchers and decision-makers responsible for environmental health policy development within Canadian Aboriginal communities; (2) explore the health researchers and decision-makers’ perceptions of the value of research evidence and Traditional Knowledge within this context and; (3) identify the preferred communication channels for disseminating and receiving research based evidence on these topics.

## 2. Methods

### 2.1. Design

The principles of fundamental qualitative description [20] were utilized to describe and understand the unique perspectives that different decision-making actors held about factors that influence environmental health KTE processes in Canadian Aboriginal communities. This type of qualitative approach has been used to provide a comprehensive summary of facts and events, using the ‘everyday’ language of the participants, and has commonly been implemented by researchers and evaluators who require answers to questions about specific events or phenomena [20]. Permission to conduct this study was received from the McMaster Faculty of Health Sciences/Hamilton Health Sciences Research Ethics Board and the University of Northern British Columbia Research Ethics Board.

### 2.2. Sample and Recruitment

To reflect the three communities theory adapted from Wingens [18], purposeful sampling was used to identify participants drawn from three distinct groups: (1) environmental health researchers (researchers); (2) external (external to Aboriginal organizations, agencies or communities) environmental health decision-makers working at Canadian Provincial/Territorial or Federal levels of government (external decision-makers); and (3) environmental health policy makers employed internally by an Aboriginal community, organization or agency (internal decision-makers). Intensity sampling, the recruitment of individuals recognized locally or nationally as experts in this field, was additionally used to ensure that in-depth and rich descriptions of KTE processes around environmental health issues impacting Canadian Aboriginal communities would be captured [21].

To achieve data saturation, we estimated recruiting a total sample of 30 individuals into the study, with 10 participants in each of the three sub-categories of participants. The inclusion criteria for the study were: (1) confirmation of experience conducting environmental health research with Aboriginal communities or employed in a role as an external or internal decision-maker involved in the development or implementation of environmental health policies impacting Aboriginal communities and; (2) the ability to speak and read English. The research team and the National Collaborating Centre for Aboriginal Health developed a database of known experts who met the study criteria. To facilitate the process of identifying and recruiting internal decision-makers, a list of key contacts for the Provincial Territorial Organizations affiliated with the Assembly of First Nations was developed and members meeting the inclusion criteria were invited to participate. Snowball sampling was also utilized whereby at the end of each interview, the study participant was invited to recommend an expert in the field who he/she perceived would have valuable experiences and information to share about KTE [21].

### 2.3. Data Collection and Analysis

Informed consent to participate in the interview was obtained from each individual participant. Each participant was invited to complete a semi-structured, in-depth interview lasting 60–90 minutes. As participants were residing in different geographical regions across Canada, the majority of the interviews (n = 26) were conducted by telephone. At the request of two participants, two of the interviews were conducted face-to-face. One participant also chose to provide written responses to the questions posed in addition to completing the interview. All interviews were digitally recorded and primary data were collected between August 2008 and May 2009.

To specifically capture the unique differences in activities conducted by researchers and decision-makers, two distinct semi-structured interview guides (Appendix A) were developed using the knowledge transfer theoretical framework developed by Lavis and colleagues [10]. Additional questions were added to explore the nature of integrating Traditional Knowledge into the decision-making processes. As the study progressed, the interview guides were adapted to facilitate the exploration of new or unique themes that were emerging such as the historical relationships between Aboriginal peoples and researchers that were characterized by mistrust and the diversity of worldviews held by different groups of individuals. Participants were also asked to complete a short demographic questionnaire. Participants were requested to share (if available) relevant documents that illustrated past or current KTE activities. Each participant was given an honorarium in the form of a $25.00 gift card for his or her participation in the interview and honorarium was not used in the recruitment process.

All recorded data were transcribed verbatim and to maintain anonymity all identifying information was removed. The principles of directed content analysis [22] guided the coding and analysis of each transcript. Initial coding categories were determined using the questions and core concepts from the interview guide. New ideas and concepts that emerged from early interviews resulted in the development of novel categories. Lists of the coding categories and sub-categories are summarized in Table 1. A brief summary of the key findings, grouped by category, was developed for each transcript. A small sample of these summaries, along with copies of the original transcripts, was sent to two co-investigators (MD, CF) so they could confirm that no key findings had been omitted from the summaries and that categories developed made sense to researchers not involved in the initial coding and categorization process. This constituted a form of verification for inter-coder variability. Finally, data coded to the categories were then synthesized by participant sub-group and compared across participant groupings.

Once data collection and analysis was completed, member checking, a technique used to promote data credibility, was undertaken between August and November 2009. In this process, used to promote data credibility, the research team’s interpretation of the interview data was shared back to the participant, who then had the opportunity to comment on the accuracy of the interpretation [23]. In order to accomplish this, the Final Report and Executive Summary were sent to all 28 participants via email. They were asked to provide feedback on the report and the summary, either through a second telephone interview or in writing, and to share their impressions of the interpretation of the data. At least two attempts (one by telephone, one by email) were made to contact each participant to ensure that all had ample opportunity to review and respond.

## 3. Results

A sample of 28 Canadian environmental health researchers (n = 10), internal (n = 9) and external decision-makers (n = 9) participated in the primary interview and of these, 13 participants completed the member checking process (3 researchers, 5 internal and 5 external decision-makers) (Table 2). While we attempted to recruit participants from jurisdictions across Canada, this sample contains individuals from six of the ten Canadian provinces. However, many study participants spoke of their experiences of working with different First Nations or Inuit populations across multiple Canadian provinces and territories. Overall, the participants had worked in their current positions for an average of 14 years. Therefore, this purposeful sample was well positioned to provide in-depth descriptions about research utilization in the field of environmental health and to provide commentary about the environmental, political and social factors influencing research and Traditional Knowledge access and utilization in the development of environmental health policy impacting Canadian Aboriginal (either First Nations or Inuit) communities.

The researchers who participated had conducted studies in a broad range of fields including: ocean science, fisheries and marine science, environmental health, risk assessments, health services, anthropology, environmental contaminants and human toxicology, pharmacology, northern climate change and contaminants, and natural resource and wildlife management. All of the external decision-makers who participated were employed at a manager level or higher within their respective departments; nine of the external decision-makers worked within a Federal agency and one external decision-maker worked within a Provincial Ministry. All of these decision-makers confirmed that they were responsible for developing or implementing either environmental health policy for First Nations or Inuit communities, conducting environmental impact assessments (EIA), or coordinating relevant national programs. Given the lessons learned around aspects of environmental health KTE (communication, knowledge translation, presentation of evidence for decision-making, and the role of Traditional Knowledge in the process) in association with many EIAs on Aboriginal lands, experts with this knowledge were included among our participants and we draw upon some of that literature in the study. The nine internal decision-makers were employed by, and working within, First Nations or Inuit communities in roles responsible for analyzing environmental health policy or implementing programs impacting environmental health outcomes.

The fundamental principles promoting the transfer and exchange of different types of evidence between environmental health researchers and decision-makers emerged from this study and included, that: (1) to facilitate successful KTE, relationships characterized by trust, respect, empowerment and equity must be initiated and nurtured; (2) KTE activities need to be negotiated early and implemented throughout the full research process and; (3) environmental health research evidence is best transferred to Aboriginal communities by crafting locally relevant messages, selecting messengers perceived as credible by the target audience and using multiple communication channels.

### 3.1. Development of Relationships in Research to Support KTE

For successful KTE to occur, relationships at all levels of decision-making must be initiated and nurtured throughout a research project. There was consensus among *all* 28 participants that researchers interested in conducting studies within Aboriginal communities must seek explicit consent from community leadership and engage community members throughout the process. It was also consistently acknowledged that this process required researcher presence in the community, which often took a lengthy period of time and required patience. It was identified that without a relationship built on trust, researchers will run significant risks of not having access to communities and may lack the necessary permissions to collect data.

Internal decision-makers highlighted that it is vital for researchers to understand that each Aboriginal community is unique and may have its own specific protocols and etiquette for the conduct of research. To facilitate community engagement, internal decision-makers identified that researchers need to establish close connections with trusted community members who can act as guides and introduce the research team to the community leadership and then seek permission to conduct research from the Chief or Band Council and other stakeholders who hold informal power such as Elders or the Council of Women. Physically travelling to Aboriginal communities, meeting face-to-face with community leadership, and introducing oneself through the sharing of both personal and professional information were identified as factors that support the development of relationships with communities. Often these early meetings are social in nature, assist in addressing preconceptions a community may hold about researchers and provide an opportunity for researchers to develop projects designed to meet community needs. One external decision-maker expressed that:

You’ve got to recognize that if you’re a non-indigenous person walking into an indigenous community, you are going to be a true outsider. You cannot just walk in and say, “Well, I have credentials.” Credentials are of interest but really not that terribly important to indigenous communities. They want to know who you are and what you’re all about. So you will have to come in and be prepared to build confidence and trust with that community before you can even start with any kind of research. It’s going to take you considerable time to build the trust to incorporate and engage their ideas into your research.

Given current funding structures, the necessity of holding these types of meetings was identified as a potential barrier for researchers, especially new investigators, who may prefer to discuss issues by telephone or email as they lack the resources to travel to a community in the early stages of project development.

It was explained that a strong relationship between researchers and Aboriginal communities provides benefits to both groups. If the community sees that a researcher is committed and willing to work for their benefit, they are more likely to provide “in kind” types of support and services. Internal decision-makers also shared that a researcher’s contribution to helping the community resolve an environmental concern will be reciprocated by community leadership promoting participation in the study and by identifying key social networks. Several of the researchers expressed hope that if mutually trusting relationships are developed with communities, then potential mistrust of researchers’ mismanagement of data will subsequently decrease. It also emerged that it is essential for these relationships to be characterized by respect, equity and empowerment.

Internal decision-makers shared that respect for the community is demonstrated through truthfully and clearly communicating the study objectives; seeking to understand the needs and worldviews of the Aboriginal community; and identifying strategies to successfully navigate through conflict. Researchers entering into communities can also establish respect by genuinely listening and learning about local concerns and identifying research questions the community needs answered.

Several decision-makers and researchers identified that one way of demonstrating equity is by valuing different types of evidence, and particularly by valuing the knowledge that is shared by the community. Through long-established relationships, the knowledge held by researchers may also come to be valued and they may be called upon by the community as consultants to provide expert opinions on emerging issues. “*The goal*”, as one internal decision-maker commented, “*is to move from having a researcher and a community to having a research community, with each player working in his area of expertise”.*

The concept of empowering communities to develop the skills, knowledge and capacity to independently conduct research about their local environments was an important theme that emerged from both groups of decision-makers. Involving community members in research may also provide much-needed employment opportunities. It was identified that when communities are empowered and hold the necessary skills to conduct research, then they can assume the leadership role to initiate and conduct local research projects. A model of research where the Aboriginal community, instead of the researcher, identifies the research questions was identified as a key strategy for addressing issues of local relevance. To facilitate this type research, internal decision-makers highlighted the importance of having knowledge of, or access to, researchers who may be interested in partnering with them to complete a project. Several participants identified the need for, or identified existing programs (e.g., First Nations Environmental Health Innovation Network http://www.fnehin.ca and ArcticNet http://www.arcticnet.ulaval.ca), that serve as bridges linking communities with environmental health questions with researchers who have the knowledge and expertise to partner in answering these questions. It was identified however, that if organizations develop researcher databases, it is important to keep them updated and to ensure that Aboriginal community leaders can access the information by the Internet and/or by connecting with a consultant over the telephone. As one internal First Nations decision-maker explained, “*I’d rather call the person I know. I’m the type of person, where I just call her up and ask for the [information]…if she knows of something that is relevant to me”.*

### 3.2. KTE Integrated into the Environmental Health Research Process

Not surprisingly, in addition to providing entry to a community and in identifying local priorities, establishing relationships with key community members was also identified by the majority of participants as a strategy for facilitating research dissemination. Decision-makers and researchers concurred that the most effective strategy for promoting the sharing, uptake and utilization of information between stakeholder groups is to negotiate KTE strategies at the beginning of a research project and to then integrate them throughout the research process. Both internal and external decision-makers highlighted the importance of not considering research dissemination to be a single act that is added on at the end of a project. It was identified that to achieve this goal, researchers need to move away from the perspective of conducting research “on” Aboriginal populations and to a perspective of working collaboratively with communities to develop and answer locally relevant research questions.

To promote the eventual uptake of evidence emerging from environmental health studies the following strategies for integrating KTE into the research process were recommended: (1) researchers invite internal decision-makers and local community members to help craft the research questions or the focus for the environmental assessment; (2) train local community members to collect data; (3) involve community members and local Elders to participate in data analysis and interpretation and; (4) ensure that there is a local community member or cultural broker who is involved in any formal dissemination strategies. As one researcher concluded:

Essentially it involves, that whomever you’re working with, be it First Nations or an organization, that they’re involved in the entire process. Hopefully, the research idea comes from their questions, and that they’re involved in the design of the study so ensuring that whatever the researchers will come up with [as findings] don’t just sit on a shelf, they’re actually used.

The full benefits of conducting collaborative research on KTE outcomes is summarized by one external decision-maker:

From the view of a classic scientific approach where relationships between the scientists and the community are not very strong there hasn’t been really a mutual, cross-pollination of approaches and perspectives and then the quality of the outcome usually is weaker. However, there are projects where researchers did take time to develop this relationship, to ask the right questions, to consult with the Elders of the communities, to consult with other power groups like women’s circle and youth groups, and really, in the set up of their projects try to understand why the community sees this topic as important. If that happens then it increases the researcher’s capacity to incorporate traditional knowledge perspectives into this research and give an opportunity to knowledge holders in the communities to actually, to provide their perspectives in the project, which actually gives a result from the projects that are quite unlike anything else that you can’t get through the traditional scientific approach. So this becomes more of an action research, it becomes more research that is up taken almost immediately after its completion by the community.

It was suggested by both researchers and decision-makers that the active collaboration of Aboriginal community members in environmental health research projects may also address existing barriers that limit the integration of research evidence into local environmental health policy. Participants identified several characteristics about the nature of scientific evidence in the field of environmental health that potentially limits its uptake in Aboriginal communities. First, some individuals and communities mistrust scientific information based on historical or past personal experiences with researchers. Secondly, it is often difficult for a community to validate information about environmental health issues, as they may be inundated with excessive amounts of conflicting information about a specific topic. Third, individuals may perceive that researchers or government agencies selectively choose to share research evidence that supports a decision or policy that has already been made. Finally, the language used in many impact or environmental assessments is often highly technical and jargon-laden, resulting in decreased comprehensibility of the findings and thus limited utility of the data. In many communities, individuals may also lack the tools and skills to effectively access research evidence and then to critically appraise it. It was noted that many remote or Northern communities still lack consistent access to high-speed Internet or computers.

### 3.3. Perceived Value of Research Evidence and Traditional Knowledge

When KTE strategies are an integral part of the research process it was explained that natural opportunities are created where Traditional Knowledge from within communities can be shared with researchers. The majority of stakeholders acknowledged that at a fundamental level, scientists and Aboriginal communities hold different worldviews about processes for “knowing the world in which we live” and the types of evidence valued in decision-making. Given the extensive intrinsic knowledge many Aboriginal people have of the land and environment, meeting the challenge of identifying processes that respect and utilize both forms of knowledge in decision-making is pivotal in environmental health discussions.

In comparing data across groups, it emerged that within government and academic departments and Aboriginal communities, there is an increasing mutual appreciation of the knowledge valued by “others.” Some researchers perceived that communities, particularly those communities with close geographic or social links to universities or long established relationships with research teams, had increasing interest in accessing and utilizing scientific evidence. One researcher shared that:

My impression after a number of years working in the [Arctic] is that things have really turned in that the value of science is recognized in the communities and the value of working with the scientific community is seen as important. Part of the reason why that’s now the case is that people have more control over their own lands and have land claims agreements and self-government agreements in some instances. So they need to know what’s going on for their own governance themselves.

From an internal decision-maker perspective, it was presented that there is value in adopting and utilizing scientific evidence in that it facilitates a community’s ability to have open dialogues with government departments, where “science” is the language most commonly spoken and understood. Some internal decision-makers identified that when attempting to mobilize government departments to respond to local environmental hazards or toxins, adopting scientific language in their communications increased the likelihood that the community’s messages would be picked up by the media. Most importantly, when action was required, scientific evidence was sometimes selectively used as powerful tool to “prove” or support the conclusions from anecdotal or community knowledge.

Based on the data from the internal decision-makers, it was determined that Aboriginal decision-makers evaluate the credibility of scientific evidence on the basis of: (1) its relevance to the community; (2) the perceived agenda of the researcher; and (3) the source of funding for the research project. It was cautioned, however, that historical research practices have resulted in many communities lacking trust in both researchers and the findings of scientific studies. As one external decision-explained:

Historically, in the past, it has been government or industry coming in with highly paid professionals with three or four degrees standing up in front of the community and saying, “We know everything, we’ve got it all worked out, here’s the science”, and they’ll show some graphs and figures and experiments. And then assume that the community will just accept it. And some communities have in the past—again indigenous and non-indigenous—and some have been hurt by that and everybody now is a little bit apprehensive when government comes in and says, “ Trust me, I’m here to help you”.

The greater cultural shift, however, is occurring amongst government and academic departments, where there is increasing acknowledgment of the value of understanding and seeking out Traditional Knowledge when working in the environmental health field. It was explained that in government policy shops, written knowledge and scientific evidence are more frequently used than oral or anecdotal knowledge. However, there was general consensus that Traditional Knowledge is valuable in identifying and refining research questions, providing cultural and spiritual insight about the phenomena under study, and providing interpretations of scientific data that make sense to the local community. It was consistently expressed by all three types of stakeholders that the two types of knowledge are different and distinct but are complementary to each other, that they should be equally valued and that use of both types of knowledge will provide a holistic understanding of environmental issues.

Researchers gaining entry into Aboriginal communities also need to have an awareness that Traditional Knowledge may be accepted and viewed by that community as more credible than the scientific data they are presenting. As one health professional in a First Nations community commented, “*You cannot go into a First Nations community or an Inuit region if you are not ready to listen to what people have to say about their own experiences and their own types of evidence”.*

While it is acknowledged that it is important to include and appreciate the importance of Traditional Knowledge, it was disclosed that the very sharing of this knowledge may create tensions between Aboriginal communities, government departments and researchers. It is essential that, if Traditional Knowledge is shared with a research team, that consent of the community or Elder who has shared his/her Traditional Knowledge be obtained prior to publicly disseminating the information and that processes to acknowledge the source of the information are established before it is disseminated. One internal decision-maker expressed that:

I can see some challenges where some of the Elders and knowledge holders don’t want to share anything anymore because they’ve been ripped off too much. So they’ve already put their wall up. They don’t want to participate.

### 3.4. Dissemination of Information

The communication of the findings from a research project or environmental assessment is still an integral component of a KTE process. Participants in this study shared their experiences and perspectives of the elements required for successfully: (1) crafting key messages; (2) locating credible messengers to deliver the messages; and (3) identifying the communication channels commonly used to disseminate information.

An important step in the KTE process is for researchers to identify what specific findings will be communicated to target audiences. Given the highly technical nature of environmental health studies, it is essential that key messages be crafted using plain language and limited jargon. It was recommended that in preparing reports, key messages be developed by synthesizing findings from multiple projects or reports and to include different perspectives about the issue facilitating decision-makers to have increased understanding of the phenomenon. In communities where the project has been conducted using participatory methods, any community concerns that were raised in the development stage of the project should be also prioritized as key messages at the end of the project.

It was recommended that researchers develop key messages that are relevant to, and resonate with, the community. One proposed strategy to achieve this goal is to deliver the key message as a story and when possible, integrate Traditional Knowledge to assist in the interpretation of findings. It was also highlighted that key messages in presentations to the community should focus on the study results and not on the scientific intricacies of the methods used to collect and analyze the data.

Given that environmental health studies are often focused on measuring toxin exposure or environmental contaminants, it was recommended that messages around risks should be developed with caution to avoid alarming community members. It is important to present a balance of both risks and benefits to certain courses of action and to examine the issue from a culturally sensitive position. Researchers should also be aware that if findings and key messages are preponderantly negative, then the community may perceive that the study will put their community in a “*bad light*” and they may choose to not permit the information to be released or may not utilize the findings. It is therefore imperative that community partners play a key role in crafting key messages. As one researcher shared:

This is best done with community partners so we always have the right language and perspective. Whenever we do the crafting ourselves the KT is less effective. Also, piloting and evaluating the effect of messages before general dissemination has proven worthwhile.

Cultural brokers, individuals who hold a personal understanding of Aboriginal beliefs, values and traditions of the community and have the knowledge and skills to interpret impact assessments or research findings, may be employed to assist researchers in crafting culturally relevant key messages. For example, one researcher shared that:

Messages need to be relayed back to the community and that’s where stakeholders, including the health authority, need to work with me, so I will put the results into the proper context.

An internal decision-maker confirmed the value of cultural brokers also by explaining:

The field workers working in the project learn how the scientific side of everything works but they also have the traditional knowledge and the knowledge of the community on the other side and know how to interpret that data. They’re a bridge between the scientific side and the community side.

Cultural brokers can also assist researchers and government decision-makers in identifying findings that may be questioned or challenged by the local community and assist them in preparing appropriate responses. As one Federal external decision-maker shared:

You cannot control the message. You may have a message but you’re going to be challenged on a lot of different fronts. So we don’t tend to just walk into a community with a small little piece of information. If we have [specific results to share with a community] we sit down and we work with AFN [Assembly of First Nations] and ITK [Inuit Tapiriit Kanatami ] and say, “Ok if we go into the community with this kind of information, what are the issues that you would think will flare up?” And then we try to get answers or bring in people who might be able to answer those kinds of questions before. We don’t tend to just walk in cold.

In addition to crafting key messages, cultural brokers may be effectively used to assist research teams in disseminating key messages. Often these cultural brokers were identified by Stakeholders as local Aboriginal health professionals, members of the Regional Contaminants Committees, a member of the local Environment Committee, or community members who had been involved in the studies. Cultural brokers are knowledgeable about how to effectively share information and where community members can be accessed. As one external decision-maker who works predominantly with First Nations populations explained:

That’s why when we communicate with First Nations and Aboriginal organizations we try to ensure that there’s someone that’s part of a team that has a trusted voice in that community to deliver information to them. So it’s not just coming from strangers that are coming from outside of the region, it’s coming from people that are trusted in that community and would basically have some understanding of the cultural sensitivity or issues that are very specific to that region that have to be taken into consideration. Issues that a general researcher or even a health practitioner would not necessarily be aware of. So it’s critical to have those types of people involved in a team approach.

One external decision-maker expressed that although cultural brokers have great value, there could be challenges in the role.

I believe this is a role that will continue to grow in demand and importance; however, it will be a challenge for many First Nations to fill such roles as they risk being criticized or ostracized for co-opting their First Nation worldview, so the value of the ‘two-eyed seeing approach’ needs to be embraced and promoted by both sides. This is the only way there will be harmonious and effective working relationships.

In many communities, researchers, particularly those who have invested in relationship development, are also viewed as credible messengers. However, it was noted that not all scientists have the skills to be effective communicators. Across several stakeholder interviews, participants talked about their positive experiences when researchers and cultural brokers worked together to share results as part of community tours or local presentations. It was explained that in some communities it is important to have the researcher present the information first-hand and be available to answer specific questions, and to have the cultural broker present to support the translation and interpretation of the messages.

When communicating the results of any environmental health project conducted in an Aboriginal community, it is essential that the results be presented first to the community in which the data were collected. The processes for communicating results back to a community should be negotiated at the start of a project and may vary from community to community. Discussions around data ownership are essential to conduct as tensions continue to exist between researchers’ priorities for publicly publishing data from studies they have received funding for and Aboriginal communities’ rights to own and control data emerging from their experiences. It is important to clarify with the community the procedures and format for communicating the information (written or oral formats), the languages that the information should be translated into, and the importance of including pictures or graphics in any written materials or oral presentations. The knowledge dissemination documents or materials developed within communities expressing environmental health messages that used vivid images and graphics of natural environments were perceived to be the most effective in communicating the intended messages.

Overall, it emerged that it is essential to use multiple different strategies to communicate a message and that face-to-face interactive dissemination strategies are more effective for transmitting information than paper reports. However, products such as websites, newsletters, or brief reports can play important supporting roles in disseminating information. All stakeholders shared examples of different communication and dissemination strategies. Other common approaches identified by participants included: radio ads or participation in radio call-in shows (particularly in Northern communities), community presentations, tours or workshops, attending relevant committee meetings and presenting a poster display or distributing flyers at a community social event. At the time of the interviews, none of the stakeholders who were interviewed had conducted or completed any evaluations of the effectiveness of the dissemination strategies.

## 4. Discussion

Findings from this descriptive qualitative study exploring perceptions and experiences of environmental health decision-makers and researchers working with Canadian Aboriginal populations indicate that the development of collaborative and respectful working relationships are essential for the successful implementation of research projects and the utilization of results. In a post-colonial framework for conducting research there is clear recognition that research conducted in partnership with Aboriginal communities is most successfully implemented when community-based approaches to research are applied (e.g., [24,25]). International guidelines for the ethical conduct of health research with Aboriginal populations recommend the use of participatory action research (PAR) approaches where power and decision-making are shared and research is conducted in a more culturally sensitive manner or models where the Aboriginal community assumes the lead in developing and implementing the research process [26–28]. It has been established that the use of participatory processes, where feasible, support the involvement of Aboriginal communities in developing and prioritizing research questions, promoting access to study participants, integrating Aboriginal knowledge, worldviews and philosophies in data interpretation and the building of community capacity to conduct research [29]. Our study findings contribute to this body of literature by identifying that these research approaches and their associated trends in Aboriginal research in Canada today are also fundamental to promoting the successful dissemination and utilization of environmental health research evidence by researchers and decision-makers.

A key element of most KTE frameworks is the development of purposeful, deliberate and collaborative partnerships between the producers and users of research early in the research process [11,30,31]. An increase in the utilization of research findings is more likely when decision-makers are genuinely involved throughout the research process [32]. Our findings indicate these relationships are best established early during the proposal development stage of the project, that the process can be time-consuming and that researchers are often strongly recommended by the Aboriginal community to travel and work face-to-face with community partners whenever possible to build a relationship based on trust and mutual understanding. To facilitate this process there needs to be increased recognition from funding agencies about the realities of conducting this type of research and the time and residency requirements that facilitate this process. Researchers would benefit by having access to research development grants with sufficient budgets to allow for travel to remote geographical regions and increased timelines that would provide time necessary for building sustainable partnerships. Increasingly, Canadian agencies funding Aboriginal health research such as the Indigenous Peoples’ Health Research Centre (www.iphrc.ca) and the Northern Contaminants Program, Indian and Northern Affairs Canada (http://www.ainc-inac.gc.ca/nth/ct/ncp/index-eng.asp) actively promote and require the involvement of local community members on research teams. Researchers also have a responsibility to genuinely reflect if they have the skills, aptitude and personal commitment to conduct research projects using collaborative approaches that emphasize community involvement and community ownership of data.

An important element of the research relationship is the opportunity to identify an individual who is able to facilitate knowledge transfer and exchange between Aboriginal communities and the research community. Participants explained that these facilitators are Aboriginals who are skilled in sharing their innate knowledge about the culture, values, beliefs and practices of their culture but who also have specialized knowledge and skills in being able to understand and interpret research methodologies and findings. Some of the study participants referred to these facilitators as cultural brokers. Cultural brokers have been defined as individuals who act as bridges or mediators between groups or individuals with different cultural backgrounds in order to influence change [33]. There is a history of using cultural brokers in the delivery of health care services to serve as liaisons between professionals and clients to manage and navigate care and to be a cultural guide for health care providers by sharing the health values, beliefs and practices of their community [34]. According to participants in our study, cultural brokers played a unique role in introducing researchers to community leadership and decision-makers, identifying environmental health issues of relevance to the community and a core role in all KTE activities such as developing culturally sensitive and relevant key messages, translating findings into the local language, sharing and integrating Traditional Knowledge to support the interpretation of the findings and actively participating in local dissemination activities. The cultural broker role described in the study is similar to the KTE strategy of using knowledge brokers as a link between researchers and decision-makers [35].

While most current mainstream knowledge broker models focus on strategies for effectively moving Western based scientific evidence into decision-making and policy development, KTE processes within the context of Aboriginal decision-making should identify strategies for cultural brokers to ethically and appropriately incorporate Western science and Traditional Knowledge to inform policy. Our study findings demonstrate that both researchers and decision-makers identify opportunities for using both research evidence and Traditional Knowledge to further their independent goals. Study participants discussed strategies for using Traditional Knowledge conceptually to provide deeper understanding or new perspectives on an issue but interestingly also referred to using scientific evidence symbolically to support claims or ideas established in Traditional Knowledge. Symbolic utilization of evidence, either Western-based research or Traditional Knowledge, involves purposefully locating and using information that supports a pre-determined position [46]. In this regard, it is important to recognize the potential therefore that scientific evidence may be utilized to either support or refute ideas emerging from Traditional Knowledge.

KTE models developed for Aboriginal decision-making environments should include purposeful processes for bi-directional sharing of different types of evidence [36]. The unique distinctions between western scientific evidence and Traditional Knowledge have been systematically described in the literature [36,37]. The field of environmental health has recently focused on and examined the processes for collecting Traditional Knowledge and using it alongside scientific research evidence [5,38,39] and identifying challenges, benefits and recommendations [40,41] of using both bodies of knowledge to address key environmental health issues. Berkes *et al.* [41] summarize that the key challenges for researchers and non-Aboriginal decision-makers in utilizing Traditional Knowledge include: processes for systematically translating and documenting the knowledge; the qualitative nature of the evidence; the spiritual and metaphorical components of the knowledge systems; and the challenge in developing tools to validate the knowledge that would be acceptable and meaningful to both groups. They conclude however that there is great value to examining traditional knowledge *in parallel* to the scientific evidence and recognizing the significant contribution of traditional knowledge and expertise.

The research dissemination strategies identified by researchers and decision-makers in this study parallel recommendations for disseminating research evidence in other fields, for example risk communication. Specifically that key messages relevant to the local community should be developed using plain language and delivered by messengers perceived as credible by the community using multiple, varied communication channels. The unique difference is that because of the significant cultural differences between non-Aboriginal researchers and Aboriginal communities, efforts to develop relationships, conduct the research and disseminate the results may take considerably more time and resources compared to other contexts. Specific to the field of environmental health, it was identified that key messages be carefully constructed to communicate risks and benefits to a community associated with a particular environment-human connection under study [42]. The utilization of Traditional Knowledge to conceptually explain or expand scientific evidence may also increase receptivity to and understanding of the findings. Additionally, researchers who are accustomed to traditional modes of disseminating research findings need to adapt new procedures that involve sharing information first with study participants and communities that contributed to the data and to discuss data ownership. Members of Aboriginal communities may not perceive that researchers hold the rights to independently present or publish findings without their permission. Within our study it was strongly recommended that negotiations about processes to disseminate findings and data ownership be conducted at the beginning of any project and that the principles of ownership, control, access and possession [43] be adhered to in respect to Traditional Knowledge and local data.

The primary limitation of this descriptive qualitative study is that only 28 participants of the estimated purposeful sample of 30 were recruited into the study despite an extended recruitment period and the use of multiple different recruitment techniques. However, amongst this sample of experts, data saturation was achieved both within and across the three stakeholder groups. This study was strengthened by the triangulation of data sources and data credibility was promoted through the application of inter-coder verification and member checking. While all participants acknowledged working in the broad field of environmental health, there was significant heterogeneity in terms of the specific issues studied or the types of environmental health policies developed. We were also unable to recruit individuals from each of the Canadian provinces and territories. However, the majority of the participating researchers and external decision-makers reported experiences working with different First Nations or Inuit populations across different regions of Canada. Given the diversity of Aboriginal populations across Canada and the uniqueness of their cultures, it is unlikely that all of the recommended KTE processes and strategies will be applicable to each individual community. However, despite the geographical variation and the different Aboriginal communities participants have worked with or for, multiple common themes emerged from this data. A strength of this study was that despite this variation their core KTE experiences were consistent. While qualitative findings are not intended to be generalizable, the present findings may be transferable to researchers and decision-makers working in collaboration with Aboriginal communities.

Based on these findings it would appear reasonable to recommend that researchers be genuine in their intentions and arrive in Aboriginal communities with the intent to conduct good science focused on improving community health outcomes. Compared to other contexts where health-related issues are studied, it appears that the importance of establishing relationships with decision-makers is of high importance and that it is essential that researchers working with Aboriginal communities understand the importance of prioritizing time and resources to the development of these relationships. Researchers are recommended to utilize community-based or PAR approaches to conduct their projects that will include community members in identifying and refining the research questions. However, if a question proposed by the community is not feasible to address, to maintain the trust of the community a researcher has a responsibility to honestly articulate what he/she can or cannot accomplish within the scope of a given project. It is recommended that researchers take time to engage with the community to explain the study objectives and be transparent in the discussion of the project’s potential risks and benefits to the community.

It was recommended by all participant groups in this study that individuals who are external to the Aboriginal community identify cultural brokers who will work with them to navigate the political and social networks in the community, assist in the development of research questions, identify strategies for genuinely involving community members in the research process and facilitate core KTE activities.

In the dissemination of research evidence, it was identified that key messages that are locally relevant, informed by multiple sources of information, balance environmental risks with other factors, written in lay language and that are integrated when possible with elements of Traditional Knowledge be developed. While it is traditional practice for researchers to create key messages in a written format, Aboriginal decision-makers emphasized the need to also utilize visual and oral communication strategies. This recommendation, coupled with potential concerns around literacy levels in some communities [44] should provide motivation for researchers to develop innovative and culturally appropriate formats for recording and disseminating key messages. In disseminating the final results of a study, ownership of the data should be discussed at the beginning of the study and priority should be given to sharing findings first with community leadership and members before it is published externally. Given the sensitivity of discussing data ownership these conversations may be best held within the context of trusting, respectful relationships that are developed over time. Furthermore, new processes for conducting Aboriginal health research are emerging with local communities contracting out research or developing local policies for research, data ownership and data sharing [45]. Finally, it is important to recognize and appreciate that although the worldviews of researchers, policy makers and Aboriginal communities are all different, valuable information and wisdom can be gained from each group.

## 5. Conclusions

This study took a descriptive qualitative approach to exploring the factors that influence the KTE process around environmental health issues in Canadian Aboriginal communities. We interviewed 28 researchers and decision-makers internal or external to Aboriginal organizations, agencies or communities. The study found results consistent with trends in community based, Participatory Action, risk communication and other research being conducted with or by Aboriginal communities and residents in Canada today. It highlighted the importance of early and ongoing efforts for relationship building activities between researchers and the Aboriginal community for enhanced KTE processes and opportunities as well as the significance of recognizing and involving both research evidence and TK information in decision processes within communities on these topics. Finally, a series of common principles for good risk communication (e.g., presenting the information openly and honestly, using trusted communicators, or speaking in plain non-discipline specific language) were identified as being common principles for good research communication to enhance KTE processes as well.

## Figures and Tables

**Table 1.** Coding categories and sub-categories.

**Primary coding categories**	**Sub-categories**
Participant role in organization	
Decision-making processes in Aboriginal communities	Types of evidence used in decision-making
Definitions of knowledge transfer and exchange (KTE)	Barriers to KTE KTE facilitators
Researcher-decision-maker relationships and collaborations	Early engagement Relationship development Introduction of researcher to the community Involvement of community in research process Building local research capacity Identification of cultural brokers
KTE process and activities	Timing of KTE activities Development of key messages Determination of credible messengers Identification of target audiences Communication and dissemination strategies Collaborations with cultural brokers Data ownership Academic expectations for researchers Evaluation of KTE activities KTE field examples
Integration of research evidence and Traditional Knowledge	Internal decision-maker perspectives External decision-maker perspectives Research perspectives
Aboriginal perceptions and worldviews	Health Environmental health Scientific evidence/research

**Table 2.** Participant demographics.

**Participant category**	**Gender**	**Mean age (range)**	**Mean years experience in current position (range)**
Researchers (n = 10)	Male n = 5 (50%) Female n = 5 (50%)	47 years (38–67)	14 (8–23)
External decision-makers (n = 9)	Male n = 7 (78%) Female n = 2 (22%)	52 years (36–65)	16 (5–38)
Internal decision-makers (n = 9)	Male n = 5 (56%) Female n = 4 (44%)	47 years (27–65)	11 (1–35)
**Total N = 28**	**Male n = 17 (61%)** **Female n = 11 (39%)**	**49 years**	**14 years**

## Appendix A. Interview guide for internal and external decision-makers

**Table:**

**Interview Questions**	**Probes**
1. Can you briefly describe your role in either the development or utilization of environmental health research and your relationship to working with Aboriginal communities?	
2. Can you discuss your experiences of how environmental health decisions or policies are made in First Nations communities?	Probe for who is involved in the decision-making process Identify different types of evidence used to inform decisions Identify factors that influence decision-making within organization
3. What types of knowledge or ‘evidence’ is valued by decision-makers within your organization?	Probe if different levels of decision-makers value different types of knowledge Probe for if there is a ‘hierarchy’ of evidence of if more value is placed on one type of evidence over another What is the process of resolution, if information from different knowledge sources is in conflict?
4. The process by which research evidence is shared and communicated with different audiences is an important step in the knowledge translation process. In your organization, how is information best shared and communicated?	
5. What factors influence the utilization of research evidence within your organization?	Probe for individual, organizational, cultural and environmental factors.
6. For researchers who produce research evidence relevant to environmental health decision-makers, how would you best advise them to share their research findings to decision-makers in Aboriginal communities or organizations?	
7. What is the solution for moving towards the goal of having both Aboriginal knowledge and research evidence inform environmental health policy impacting Aboriginal populations?	

## Appendix B. Interview guide for environmental health researchers

**Table:**

**Interview Questions**	**Probes**
1. Can you briefly describe your role conducting environmental health research and your relationship to working with Aboriginal communities?	
2. Can you describe your current understanding of what such terms as knowledge translation or knowledge transfer and exchange mean?	
3. Please describe at least one environmental health research project that you have participated in that involved some aspects of knowledge transfer and exchange with Aboriginal decision-makers, communities or organizations.	Probe for timing of KTE activities. Probe for how ‘key messages’ were developed. Is there a process for identifying and then involving Traditional Knowledge with the research evidence findings? Probe for process by which target audience is defined? When working with Aboriginal decision-makers or organizations, who do you perceive is a credible ‘messenger’ to share research evidence with the decision-maker partners?
4. How do you engage your target audience in the research process?	When are members of the target audience invited to participate in the research process e.g., at stage of question development, through study implementation, only at dissemination stage? What dissemination strategies have you commonly used to transfer research knowledge? What channels of communication have you used to transfer research knowledge? Can you describe what would be the most effective dissemination strategies for communicating scientific research evidence about environmental health issues to Aboriginal decision-makers? Probe for any current barriers to using what they would perceive as most ‘effective’ strategy.
5. Please describe any evaluation efforts undertaken to evaluate the effectiveness of your KTE strategies.	
6. What advice would you give to a researcher interested in collaborating with a decision-maker in an organization or community concerned about environmental health issues impacting Aboriginal communities?	
7. What is unique about the process of knowledge transfer and exchange within Aboriginal communities or organizations?	
